# The Effect of Contrast Water Therapy on Dehydration during Endurance Training Camps in Moderate-Altitude Environments

**DOI:** 10.3390/sports11120232

**Published:** 2023-11-22

**Authors:** Takayuki Inami, Shota Yamaguchi, Takuya Nishioka, Kenta Chida, Kosaku Hoshina, Osamu Ito, Takeshi Hashimoto, Mitsuyoshi Murayama

**Affiliations:** 1 Institute of Physical Education, Keio University, 4-1-1, Hiyoshi, Yokohama 223-8521, Japan; 2 Graduate School of System Design, Management, Keio University, 4-1-1, Hiyoshi, Yokohama 223-8521, Japan; 3 Graduate School of Media and Governance, Keio University, 5322, Fujisawa 252-0883, Japan; 4 Sports Medicine Research Center, Keio University, 4-1-1, Hiyoshi, Yokohama 223-8521, Japan; 5 FOCS Inc., 1-3-47, Nakahara-Ward, Kawasaki 211-0025, Japan

**Keywords:** bioimpedance analysis, titin N-terminal fragment, jump performance

## Abstract

The effects of contrast water therapy (CWT) on dehydration at moderate altitudes during training camps remain unknown. We hypothesized that CWT reduces dehydration resulting from training at moderate altitudes and improves performance, akin to conditions at sea level. A 13-day endurance training camp was held at a moderate altitude of 1100 m and included 22 university athletes, who were divided into two groups (CWT group, n = 12; control (CON) group, n = 10). The sample size was calculated based on an α level of 0.05, power (1 β) of 0.8, and effect size of 0.25 based on two-way ANOVA. Longitudinal changes over 13 days were compared using a two-group comparison model. Additionally, 16 athletes participated in an additional performance verification analysis. Subjective fatigue, body mass, and water content (total body water (TBW), extracellular water (ECW), and intracellular water) were measured using bioimpedance analysis every morning, and the titin N-terminal fragment in urine (UTF) was measured as an index of muscle damage. For performance verification, 10 consecutive jump performances (with the reactive strength index (RSI) as an indicator) were evaluated as neuromuscular function indices. The results indicated that the UTF did not significantly differ between the two groups. Moreover, the ECW/TBW values, indicative of dehydration, on days 4 and 5 in the CWT group were significantly lower than those in the CON group. However, there was no significant difference in RSI between the two groups. Therefore, although CWT reduces dehydration in the early stages of the training camp, it may not affect performance.

## 1. Introduction

To improve performance and conditioning, individual and team athletes often train at high- and/or moderate-altitude environments (e.g., training camps). However, in the early stages of moving to high and moderate altitudes, various physiological and pathological responses occur in the body, including temporary dehydration due to altitude-induced diuresis [1]. This response signifies an early environmental adaptation, emphasizing the importance of hydration [2,3,4], which cannot be overlooked given the serious impact that dehydration has on performance (e.g., endurance and sprint/jump performance). Therefore, for a successful training camp, the staff managing the training camps at high altitudes should track and accurately manage changes in body water due to dehydration [1] over time and plan for early recovery.

Previous assessments of body water content have reported that skeletal muscle damage, including exercise-induced muscle damage (EIMD) resulting from activities such as long-distance training and marathons, may change body water content and the accuracy of evaluation methods [5]. Muscle damage after endurance training and marathons has two main effects: damage that changes the structure of muscle fibers and fascia and increased intramuscular pressure due to changes in water content [5,6]. These factors sometimes act independently or interact with each other to influence the extent of muscle damage. By separately assessing the structural changes and changes in water content caused by EIMD in muscle, it is possible to clarify the recovery method that should be implemented after training.

Although muscle biopsy is the gold standard at the cellular level for the assessment of EIMD [7,8], which negatively affects muscle strength and stiffness, indirect biomarkers such as blood creatine kinase and urinary titin N-terminal fragment (UTF) are increasingly being utilized [9,10,11,12]. Notably, UTF specifically responds to eccentric contractions (exercise) related to EIMD and detects titin leakage in the urine caused by EIMD [13]. Hence, its assessment is an excellent method in competitive sports because it is non-invasive and can be performed repeatedly [9,10]. In addition, UTF also correlates with changes in muscle strength and stiffness caused by EIMD, as well as with scores on the visual analog scale (VAS), a valid measure of pain intensity [9,14]. Therefore, UTF can be used to quantify skeletal muscle damage. However, biomarkers such as blood creatine kinase and UTF are not suitable for immediate feedback in environments at high altitudes, as measurement results require several days to process [9].

In contrast to the use of biomarkers, bioimpedance analysis (BIA), which can provide immediate feedback, has also been recently used for the evaluation of EIMD. BIA is a traditional method that consists of the application of a small electric current to the body and the measurement of electrical characteristics such as resistance and impedance. This method is also a non-invasive method used not only for analysis of body composition [15] but also for monitoring nutritional and hydration status. Only recently has BIA been utilized for vector analysis after muscle damage [16] and for estimating body water content, including extracellular, intracellular, and total body water (ECW, ICW, and TBW, respectively). Shiose et al. reported that the ECW content increases at the onset of EIMD and that peak ECW content and peak creatine kinase are positively correlated [17]. In addition, it has also been understood that an increase in ECW/TBW without a decrease in ICW indicates an increase in ICW and ECW content due to vasodilation and increased permeability [18]. These findings suggest the potential for enhancing our understanding of dehydration and EIMD by analyzing water balance using BIA, which can provide immediate feedback, and by quantifying muscle damage by EIMD using UTF.

Contrast water therapy (CWT) and alternating cold and warm water immersion are used by several athletes to recover from the symptoms of EIMD. The benefits of CWT are related to a reduction in muscle soreness and improvement in muscle function compromised by the attenuation in muscle strength and power loss after exercise [19,20]. Furthermore, CWT may reduce edema by alternating peripheral vasoconstriction and vasodilation [21]. The above reports suggest that CWT could potentially be effective in addressing both dehydration and EIMD, thus potentially resolving the above-mentioned problems arising in the early stages of training camps for athletes. However, it should be noted that these findings were obtained under conditions on level ground at sea level (below; flat ground). Collectively, there is a lack of conclusive evidence regarding the impact of CWT at high altitudes on dehydration and EIMD.

Although running time is commonly measured as an evaluation of endurance performance, field-based running includes repeated accelerations and decelerations that occur during a driving test, as well as environmental influences including climate and altitude [22]. Therefore, in long-term training camps, there is a need for a method to eliminate these factors as much as possible and evaluate endurance performance in the same environment. The reactive strength index (RSI) is a metric used to examine the capacity to effectively utilize the stretch shortening cycle [23] and can provide useful information during routine monitoring of athletes about performance [22]. It has been reported that RSI and measures of endurance performance were positive and moderate, and individuals with larger RSI scores could achieve greater endurance performance, either through a reduced energy cost or greater total distance covered [22]. However, regarding the use of CWT for performance enhancement, there are also reports that CWT does not affect performance such as jumps associated with RSI [24,25]. Moreover, it must be noted that these results were also obtained under conditions on flat land, similar to those obtained in the above CWT research.

In consideration of the information above, we conducted an experiment to test the hypothesis that CWT can mitigate dehydration resulting from training at altitude. This evaluation was designed to parallel the conditions studied in prior research conducted on flat ground [19,20,21]. However, in the context of EIMD, one’s water volume can temporarily increase due to edema, making the effects on dehydration less distinguishable. Therefore, we focused on verifying the effect of CWT on dehydration at moderate altitudes during endurance training camps by limiting the training content to endurance exercises, which are unlikely to cause EIMD, and by monitoring their effects using UTF (whether the strength was adequate to cause protein leakage). As an additional verification, we assessed the impact of CWT on performance changes at moderate altitudes under the assumption that it does not affect performance.

## 2. Materials and Methods

### 2.1. Participants

Participants were openly recruited from the long-distance runner section of the track and field club of our university, and a total of 23 (all male) university athletes were included in this study. The sample size was calculated based on an α level of 0.05, power (1 β) of 0.8, and effect size of 0.25 based on two-way ANOVA. The participants were Japanese second-league-level university athletes. A 13-day training camp was held at the National Training Center facility at a moderate altitude of 1100 m (Bodaira Athlete Village, Yamagata Prefecture, Japan), during which data were collected with full participation and consent from all athletes. The participants were randomly divided into two groups (CWT (n = 12) and control (CON); n = 11), and longitudinal changes over 13 days were tracked and compared using a two-group comparison model. Unfortunately, one participant in the CON group dropped out due to illness. Finally, the CWT (n = 12) and CON (n = 10) groups of athletes were analyzed (Table 1). 

Additional validation of the performance test was performed only for the consenting participants within each group. A total of 16 athletes from the CWT and CON groups participated in the additional performance test. The characteristics of each group, including the groups that participated in the additional performance test, are shown in Table 1. Throughout the duration of this study, the participants refrained from consuming anti-inflammatory pills and did not use any additional methods to aid in recovery, such as massage.

### 2.2. Study Design

All participants underwent the same training; ate the same food for breakfast, lunch, and dinner during the camp; and bathed at a designated time after the training (in the late afternoon). During the training camp, the following items were measured at the same time every day (total, 13 points). All participants were habituated to each measure through normal sports-specific testing and training procedures within the institution. Subjective fatigue, body mass, and water content (TBW, ECW, and ICW, respectively) were measured using BIA as an index of dehydration every morning. Additionally, UTF was measured as an index of the presence of EIMD. Regarding subjective fatigue and RSI, data collection commenced from the second day (Day 2), as the first day was a transition day. In the additional performance test, 10 consecutive jump performances (with the RSI as a measure) were assessed as neuromuscular function indices (performed between the morning and evening training sessions).

### 2.3. Subjective Fatigue

Based on previous studies [9,14], subjective fatigue was expressed on a 10 cm VAS, where 0 cm indicated “comfortable, with no fatigue” and 10 cm corresponded to “too exhausted to do anything”. These subjective fatigue data were collected starting on the second day because the first day was a transition day. Thus, subjective fatigue measurements were made a total of 12 times.

### 2.4. CWT Intervention

CWT is defined as an alternating cold- and hot-water immersion [19,26,27,28]. Based on previous studies, the CWT group repeated one set of cold- (15 °C; 90 s) and hot- (42 °C; 90 s) water immersion sessions for a total of five sets (total of 15 min). In contrast, the CON group underwent hot-water immersion only in Japan (42 °C) for a similar duration (total of 15 min). During the training camp, participants were instructed to bathe in the same time zone and soak themselves up to their shoulders.

### 2.5. Endurance Training Volumes during the Training Camp

All subjectsadhered to their club training schedules during the camp. All training was conducted on mountain slopes at an altitude of approximately 1100 m. This included a daily 12 km run, three sessions of free-running (on the first day and last day of the training camp, and once midweek), 15 km of jogging, two 25 km cross-country runs, 12 sets of 1 km cross-country runs, 15 sets of 500 m runs, 12 sets of 1000 m runs, and a 16 km run followed by 1000 m of running (with lap times averaging around 3′20″ to 3′15″ on a 400 m course). The training menus are listed in Table 2.

### 2.6. BIA

The participants wore light athletic clothing and were instructed to remove shoes and any plastic, metal, or jewelry from their bodies. A multifrequency BIA device (InBody770, InBody Japan Inc., Tokyo, Japan) was used. The participants stepped on the multifrequency BIA device, held onto the handrail probe bilaterally, and remained on the device for 2 min. The analyzer uses an alternating current of 250 mA to assess TBW and ECW. The ICW was calculated using the following formula:

ICW = TBW − ECW

The multifrequency BIA device measured segmental impedances on the right arm, left arm, right leg, and left leg at the three mentioned frequencies. In this study, ECW, ICW, TBW, and ECW/TBW were used as indicators of dehydration for further analysis [16,18].

### 2.7. UTF Excretion Assay

Approximately 3 mL of urine was collected from each participant to measure the UTF concentration using an enzyme-linked immunosorbent assay (ELISA) kit (Titin N-terminal Fragment Assay Kit, Immuno-Biological Laboratories Co. Ltd., Gunma, Japan), in accordance with a previous study [29]. Samples were stored at −20 °C for later analyses. Thawed urine samples were diluted from 1:5 to 1:500 ratios to ensure that they were within the linear detection range. Diluted samples and standard solutions were added to each well of 96-well microplates coated with antibodies. Subsequently, the microplates were incubated at 37 °C for 60 min. Afterward, the microplates were washed four times with a wash buffer, and labeled antibodies were added to each well. Following this, the microplates were incubated again at 37 °C for 30 min. 

Following another five washes with the wash buffer, the microplates were incubated with tetramethylbenzidine solution at room temperature (20~25 °C) for 30 min. The stop solution was added to each well during the final step of the ELISA. Absorbance was measured at 450 nm using a microplate reader (Multiskan FC, Thermo Fisher Scientific, Tokyo, Japan). The UTF concentration was calculated using a linear regression model, and urinary creatinine levels were estimated using an automated analyzer (Bio Majesty JCA-BM8060, JEOL, Tokyo, Japan). The UTF values were normalized relative to urinary creatinine (each raw data point in the urine/Cr concentration) [29]. Notably, the normal range for UTF concentration in the general population is 1.47–7.14 pmol/mg/dL [13].

### 2.8. RSI Measurement

To measure the RSI as an indicator of performance, the participants performed 10 maximal rebounds. Upon landing from the initial jump, the participants were required to consecutively perform rebound jumps, with instructions to maximize their jump height and minimize their ground contact time. The jump heights and ground contact time were averaged across 10 rebound efforts and used to calculate the RSI as follows:

$$\mathrm{RSI}=\frac{jump\;height\;(m)}{ground\;contact\;time\;(s)}$$

Based on the RSI calculation, 10 mean RSI values were calculated for further analysis [30,31]. A custom jump mat (S-CADE. Corp. Ltd., Tokyo, Japan) was used to acquire the jump height and ground contact time data.

### 2.9. Statistical Analysis

Values are expressed as means ± standard deviations. Mauchly’s sphericity test was employed to assess the homogeneity of covariance prior to conducting the analysis of variance (ANOVA). The changes in total work during each exercise set were compared between the groups (CWT vs. CON) using a two-way ANOVA with two factors (condition × time). If a significant interaction effect was found, a post hoc test was performed to identify the time points with significant differences between the groups using Bonferroni’s method. Cohen’s d was calculated by subtracting the mean of the hot-water-immersion-only group from the mean effect of the contrast water therapy group and dividing by the pooled standard deviation where there was a statistically significant difference in the two-way ANOVA for both groups:

$$d=\frac{\bar{x_{CWT}}-\bar{x_{CON}}}{SD}$$

All statistical analyses were conducted using Predictive Analytics SoftWare (PASW) version 27.0 for Windows (SPSS Japan Inc., Tokyo, Japan). Statistical significance was set at *p* < 0.05.

### 2.10. Ethics

The study protocol was approved by the Human Research Ethics Committee of the university (approval number 23-005), and all procedures in this study were performed in accordance with the principles outlined in the Declaration of Helsinki. All participants were fully informed of the procedure and the purpose of this study, and they provided written informed consent.

## 3. Results

**Figure 1:**
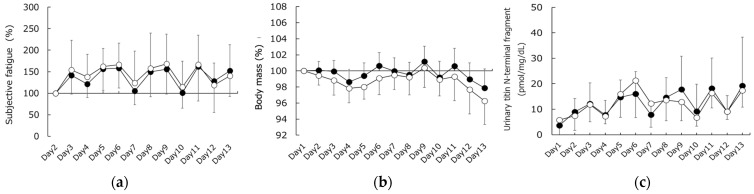
Changes in (**a**) subjective fatigue, (**b**) body mass, and (**c**) urinary titin N-terminal fragment levels. Closed circles represent the contrast water therapy (CWT) group values, while open circles represent the control (CON) group values.

**Figure 2:**
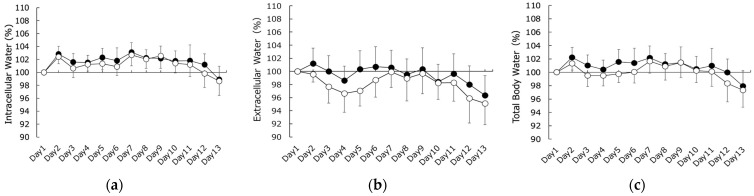
Changes in (**a**) intracellular water (ICW), (**b**) extracellular water (ECW), and (**c**) total body water (TBW) content. Closed circles represent the contrast water therapy (CWT) group values, while open circles represent the control (CON) group values.

No significant differences were observed in subjective fatigue, body mass, or UTF between the CWT and CON groups (Figure 1a–c). In addition, no significant differences in ICW, ECW, or TBW were observed between the CWT and CON groups (Figure 2a–c).

**Figure 3:**
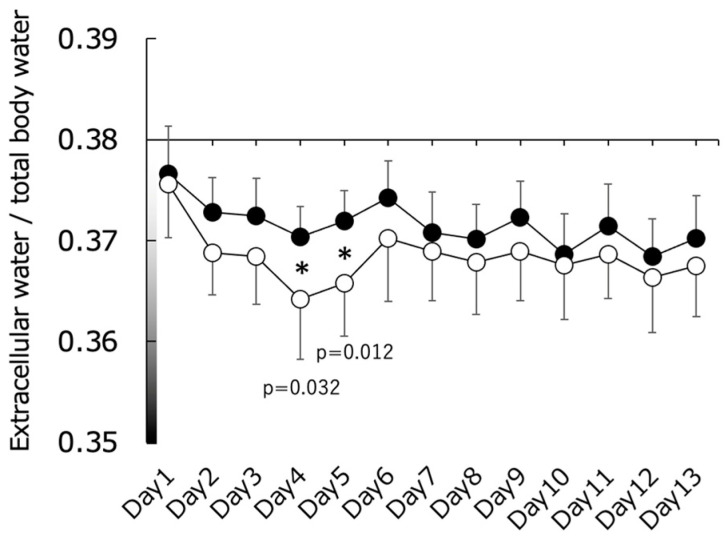
Changes in extracellular water (ECW)/total body water (TBW) content. Closed circles represent the contrast water therapy (CWT) group values, while open circles represent the control (CON) group values. Asterisks indicate statistically significant differences.

**Figure 4:**
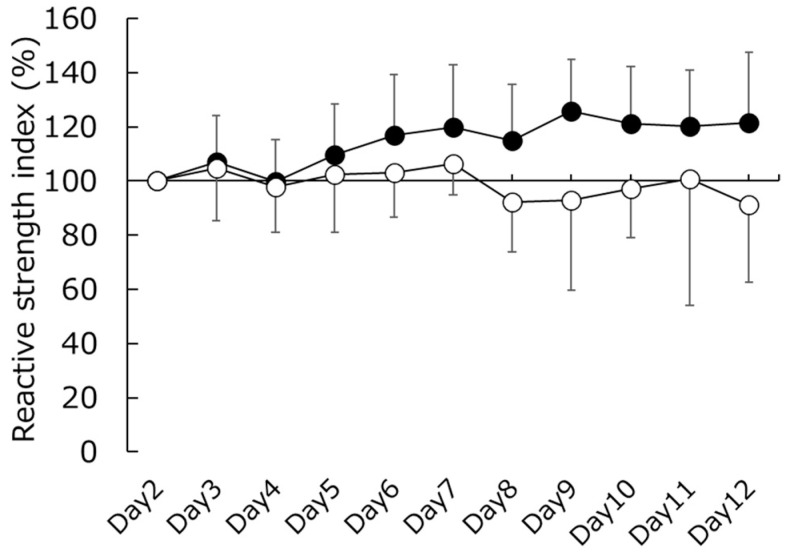
Changes in reactive strength index (RSI). Closed circles represent the contrast water therapy (CWT) group values, while open circles represent the control (CON) group values.

The results of the comparison between the CWT and CON groups for cell water content are shown in Figure 3. Specifically, the ECW/TBW for the CWT group exceeded that of the CON group on days 4 (*p* < 0.05, d = 1.066) and 5 (*p* < 0.05, d = 1.140). However, no significant difference in RSI was observed between the CWT and CON groups (Figure 4).

## 4. Discussion

We conducted an experiment to test the hypothesis that CWT reduces dehydration resulting from endurance training camps at moderate altitudes, similar to previous findings on flat ground. We found that the UTF did not significantly change between the two groups (CWT and CON) in the training camp. In addition, ECW/TBW values indicating dehydration on days 4 and 5 in the CWT group were significantly lower than those in the CON group, indicating reduced dehydration. Moreover, there was no significant difference in RSI as a performance index between the two groups (CWT and CON). These results support our hypothesis.

From the start of the training camp, the UTF repeatedly increased and decreased in both groups (Figure 1c). Subsequently, the UTF value did not return to its initial value until the last day of the training camp. This pattern of time-course changes supports the results of our previous study [9]. Intense exercise strains individual muscle fibers, resulting in the destruction of muscle cell membranes and myofibrils [32]. This destruction is observed immediately after exercise, and several hours later, the myofibrils are further degraded by proteases [32]. Calpain 3, a protease, is primarily expressed in skeletal muscle and is associated with titin [33]. Calpain 3 specifically cleaves and degrades titin, thereby inducing muscle damage, especially after eccentric contraction [7]. Consequently, the delayed peak of UTF is strongly influenced by the presence of calpain, an enzyme known for its degrading action. However, when UTF is used to assess EIMD after eccentric contractions using dumbbell exercises [9], its value increases by up to 68-fold. In the present study, UTF showed a 2-fold increase in the CON group and a 2.1-fold increase in the CWT group. These findings demonstrate that the changes observed in this study are quite small compared to those reported in previous research. Furthermore, despite the limitation of unconditional comparisons due to different movement and contraction patterns, the EIMD resulting from exercise loads in this training camp was minor, indicating that the effect of EIMD-induced swelling and edema (i.e., dehydration) on the results of this study was small.

As shown in Figure 3, the ECW/TBW ratio, an indicator of dehydration in the CON group, decreased at the beginning of the training camp and peaked on the 4th day. This indicates the temporary dehydration that occurs during the early stages of migration to high altitudes, which has been reported in previous studies [1,2,3,4]. In contrast, the CWT group also showed a decrease at the start of the training camp; however, this decrease was notably less pronounced than that observed in the CON group. This suggests that CWT suppresses dehydration during the early stages of training. CWT may reduce edema by alternating peripheral vasoconstriction and vasodilation, a mechanism referred to as the “pumping action” [21]. Although altitude-induced diuresis occurs at an early stage of acclimatization, altitude exposure has been suggested as a risk factor for hypercoagulation [34,35]. Thus, this phenomenon includes several factors besides dehydration (e.g., hypoxia, hemoconcentration, and cold temperatures). However, an increase in ECW/TBW without a decrease in ICW was reported to indicate an increase in ICW and ECW content due to vasodilation and increased permeability in a previous BIA study [18]. Since the ICW in this study did not change in either group, as shown in Figure 1, the changes in ECW/TBW in the CWT group indicated that CWT had a more positive effect on dehydration at moderate altitudes compared to normal bathing. Importantly, while dehydration did exhibit some recovery afterward, it remained at a lower level, indicating that the effect of CWT might be primarily confined to the early stages of the training camp at moderate altitudes.

In this study, we used the RSI as a performance indicator. Maximal rebounding abilities, as measured using indices such as the RSI, are impaired following prolonged running and are associated with muscle damage [30]. However, several studies showed that CWT does not improve performance [24,25]. Therefore, we tested the hypothesis that CWT at moderate altitudes does not improve performance. As a result, no significant differences were observed between the CWT and CON groups. According to Nicol et al. [30], the maximal rebound from a drop jump is associated with increased impact peaks and extended ground contact times. This implies that the RSI reduction is attributable to muscle damage. Oliver et al. [31] also reported that RSI values do not contribute to the accumulation of long-term fatigue; thus, the finding that the mean RSI of both groups did not decrease in this study is considered reasonable, especially considering the UTF results (which confirmed no/little muscle damage in this training camp). In summary, we found that CWT at moderate altitudes did not result in an improvement in RSI performance (no change). It is necessary to consider that previous CWT studies were conducted on flat ground. Additionally, in this study, the final CWT session involved cold water. The physiological effects of cold water include decreased nerve conduction velocity and excitability [36] and reduced neural (nociceptive) transmission [26]. Cold-water application may lead to reduced blood flow to the muscles [37], potentially resulting in decreased edema and a reduction in the initiation of inflammatory events. However, the extent to which these effects influence performance remains inadequately understood, and it is worth noting that concluding CWT with immersion in cold water has been a common practice in previous reviews [20]. Based on our results, it is difficult to discuss the impact of CWT and cold water in the final CWT session on performance at moderate altitudes in detail. While the restorative benefits of CWT in mitigating EIMD are undeniable, its impact on moderate-altitude performance in the absence of EIMD requires further study.

We found that CWT at a moderate altitude had a more positive effect on dehydration in the early stage of the training camp compared to normal bathing. However, when training camps are planned for short rather than long periods, the risk that the training camp schedule will be prioritized should be considered (i.e., without allowing sufficient time for adaptation, resulting in immediate intense training post arrival). In this context, our study provides new insights into the effects of CWT at moderate altitudes. CWT may be an effective tool for conducting better training camps at moderate and/or high altitudes, leading to better performance and conditioning control. However, while the effectiveness of CWT has been well established in flat ground environments, its efficacy in altitude settings, including moderate altitudes, remains to be verified. Based on findings in conventional settings, the recovery effect of CWT after EIMD is nearly equivalent to those of widely practiced techniques such as stretching [20]. Moreover, muscle strength and power loss resulting from EIMD also tend to recover more rapidly after CWT compared to no intervention. A verification that combines these methods and CWT at moderate and/or high altitudes is required in the future.

This study had some limitations. First, this study was conducted at a moderate altitude of 1100 m. However, it is unknown whether this would have the same effect at higher elevations. Secondly, this study used a two-group comparative design. Therefore, it should be noted that the crossover effect could not be verified.

## 5. Conclusions

In this study, we found that CWT at moderate altitudes had a more positive effect on dehydration in the early stages of the training camp compared to normal bathing. However, it should be noted that this CWT effect at moderate altitudes might be limited to the early stages.

## Figures and Tables

**Table 1:** Characteristics of each group.

Additional Performance Test						
	n	Age (Years)	BMI (kg/m^2^)	n	Age (Years)	BMI (kg/m^2^)
Contrast water therapy group	12	19.7 ± 0.9	20.0 ± 1.9	8	20.0 ± 1.1	20.2 ± 2.1
Control group	10	20.3 ± 1.2	20.2 ± 1.7	8	20.1 ± 1.0	20.0 ± 1.9

**Table 2:** Endurance training volumes during the training camp.

	Early Morning	Late Morning	Late Afternoon
Day 1	Transfer		15 km JOG
Day2	12 km RUN	15 km JOG + 3–5 × 200 m strides run	CT + 40 min − JOG
Day 3	12 km RUN	15 km JOG + 3–5 × 200 m strides run	Free
Day 4	12 km JOG	20–25 km XC	CT + 40 min − JOG
Day 5	12 km RUN	15 × 500 m (R500 m JOG)	CT + 40 min − JOG
Day 6	12 km RUN	Free	Free
Day 7	12 km JOG	20–25 km XC	CT + 40 min − JOG
Day 8	12 km RUN	10–12 × 1 km XC (4′)	CT + 40 min − JOG
Day 9	12 km RUN	Free	Free
Day 10	12 km JOG	12–15 × 1 km (R200 m JOG) 3′10″-3′00″	CT + 40 min − JOG
Day 11	12 km RUN	Free	Free
Day 12	12 km RUN	16 km + 1 km (R400 m JOG) 3′30″-3′15″	CT + 40 min − JOG
Day 13	15 km JOG	Transfer	

JOG: jogging, RUN: running, XC: cross-country, CT: core training.

## Data Availability

The data that support the findings of this study are available from the corresponding author upon reasonable request.
